# Spontaneous Peritoneal Hemorrhage and Anemia: A Rare Case Report of Immune Thrombocytopenic Purpura

**DOI:** 10.7759/cureus.64602

**Published:** 2024-07-15

**Authors:** Diana M Almeida, Duarte Silva, Marta Henriques, Ricardo C Costa, Joana Azevedo

**Affiliations:** 1 Pediatrics, Hospital Distrital da Figueira da Foz, Unidade Local de Saúde do Baixo Mondego, Figueira da Foz, PRT; 2 Clinical Hematology, Unidade Local de Saúde de Coimbra, Coimbra, PRT; 3 Hematology and Medical Oncology, Unidade Local de Saúde de São João, Porto, PRT; 4 Pediatrics, Unidade Local de Saúde de Coimbra, Coimbra, PRT

**Keywords:** clinical case, rare presentation, anemia, peritoneal hemorrhage, immune thrombocytopenia

## Abstract

Immune thrombocytopenia (ITP) is characterized by isolated thrombocytopenia manifesting with mucocutaneous bleeding symptoms, generally mild to moderate. The presence of severe symptoms or complications is rare but can be life-threatening and should be promptly diagnosed and treated.

We present the case of a 14-year-old female presenting with abdominal tenderness and signs of peritoneal irritation and found to exhibit petechial rash in the buccal mucosa, scant petechiae, and superficial ecchymosis in both arms and legs on physical examination. Laboratory evaluation revealed severe thrombocytopenia and normocytic anemia. Abdominal ultrasound showed a significant peritoneal hematic effusion. The diagnosis of ITP with spontaneous peritoneal hemorrhage was made, and she was treated with intravenous immunoglobulin (IVIG) and antibiotic therapy, as well as one packed red blood cell transfusion because of worsened anemia on re-evaluation. A gradual rise in platelet count and hemoglobin was observed, as well as a gradual resolution of the peritoneal hemorrhage, with no further therapy.

## Introduction

Immune thrombocytopenia (ITP) is the most common acquired bleeding disorder in childhood (estimated annual incidence of one to 6.4 cases per 100000 children), characterized by isolated thrombocytopenia [1-3]. The typical presentation is the sudden appearance of mild to moderate mucocutaneous bleeding, with no other accompanying symptoms, in an otherwise healthy child or adolescent [1,2,4]. The prognosis is usually very good, with rates of 80% to 90% of complete remission in the first six months after disease onset, irrespective of the treatment [2,3,5,6]. Severe bleeding, such as mucosal bleeding or internal hemorrhage (e.g., severe gastrointestinal or genitourinary bleeding requiring immediate attention and intervention), and other potentially life-threatening conditions (e.g., intracranial hemorrhage) are rare. Nevertheless, they are major causes of mortality and should be promptly diagnosed and treated [1,3-5]. In this case report, we present the case of a 14-year-old ITP patient with a spontaneous peritoneal hemorrhage and anemia.

This article was previously presented as a Poster at the 2º Congreso Ibérico de Hematología y Oncología Pediátricas on May 25-27, 2023.

## Case presentation

A 14-year-old female was brought to the emergency department with a 24-hour history of constant and dull abdominal pain in the hypogastric area, with a gradual onset and progression, with no migration, little improvement with paracetamol, and night awakening because of the pain. She had no fever or other gastrointestinal or genitourinary symptoms and denied previous trauma.

She had been diagnosed with acute tonsillitis three weeks earlier (treated with Amoxicillin and nonsteroidal anti-inflammatory drugs), but was otherwise healthy, with no chronic medications. Her menstrual cycle was regular at 28 to 30 days, and her last menstrual period had been four weeks before.

She was hemodynamically stable, with blood pressure of 98/57 mmHg, heart rate of 91 bpm, no fever, good general appearance, and no evidence of neurological impairment. No active bleeding was seen or reported (including no menstrual losses), but the physical examination was relevant for the presence of petechial rash in the buccal mucosa, scarce petechia, and superficial ecchymosis in both arms and legs, which had not been recognized as relevant by the girl and family, as well as abdominal tenderness with signs of peritoneal irritation. No palpable hepatosplenomegaly or lymphadenopathies were described.

**Figure 1:**
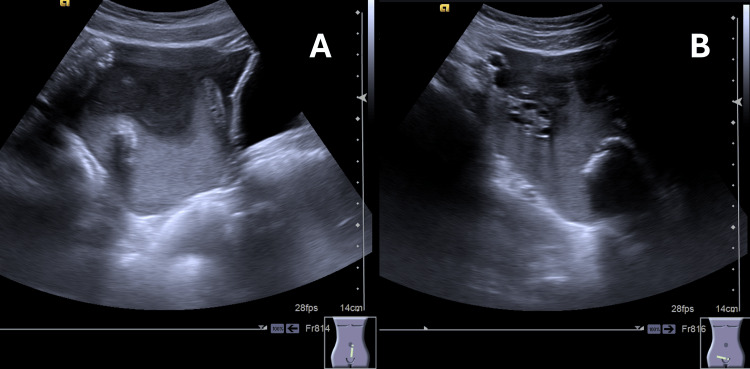
Abdominal ultrasound revealing significant free hematic peritoneal effusion

Laboratory evaluation (Table 1) revealed severe thrombocytopenia (4x10^9^/L) and normocytic anemia (hemoglobin: 8.7 g/dL, mean corpuscular volume (MCV): 91 fL) with normal reticulocyte count and normal white blood cell count (7.5x10^9^/L). Blood smear showed no evidence of erythrocyte fragmentation or immature cells. General chemistry and coagulation evaluation were unremarkable, the Coombs test was negative, and ADMTS13 activity was 66%. Epstein-Barr virus (EBV) serology was suggestive of recent infection. The pregnancy test was negative. Abdominal ultrasound, presented in Figure 1A, 1B, revealed significant free hematic peritoneal effusion, with no other remarkable findings. The source of the bleeding was not identified.

**Table 1:** Initial laboratory evaluation at first presentation ALT: alanine transaminase; anti-HBc: antibody to hepatitis b core antigen; anti-HCV: antibodies to hepatitis c virus; anti-HIV: antibodies to human immunodeficiency virus; aPTT: activated partial thromboplastin time; AST: aspartate aminotransferase; CRP: C-reative protein; BUN: blood urea nitrogen; H: high; HBsAg: hepatitis b surface antigen; L: low; MCH: mean corpuscular hemoglobin; MCHC: mean corpuscular hemoglobin concentration; MCV: mean corpuscular volume; N: normal; RDW: red blood cell distribution width; WBC: white blood cell; EBV: Epstein-Barr virus

Laboratory test	Patient’s results	Reference range	Interpretation
Hematology			
WBC count	7.5x10^9^/L	4.5-13.0x10^9^/L	N
Neutrophile	4.3x10^9^/L	1.8-8.0x10^9^/L	N
Lymphocyte	2.5x10^9^/L	1.5-5.2x10^9^/L	N
Monocyte	0.4x10^9^/L	0.1-1.0x10^9^/L	N
Eosinophil	0.06x10^9^/L	0.02-0.65x10^9^/L	N
Basophil	0.1x10^9^/L	0.0-0.2x10^9^/L	N
Erythrocytes	2.8x10^12^/L	4.1-5.1 x10^12^/L	L
Hemoglobin	8.7 g/dL	12.0-16.0g/dL	L
Hematocrit	25.1%	36-46%	L
MCV	91 fL	78-102 fL	N
MCH	31.2 pg	25-35 pg	N
MCHC	34.7 g/dL	31-37 g/dL	N
RDW	13.4	11.5-15.0	N
Platelets	4x10^9^/L	160-400x10^9^/L	L
Reticulocyte count	92x10^9^/L	30-105x10^9^/L	N
Blood chemistry			
BUN	10.5 mg/dL	7-20 mg/dL	N
Creatinine	0.58 mg/dL	0.57-0.80 mg/dL	N
AST	38 U/L	<31 U/L	H
ALT	39 U/L	<34 U/L	H
CRP	0.05 mg/dL	<0.50 mg/dL	N
Coagulation			
Prothrombin time	14.5 s	10.0-14.1 s	H
aPTT	32.0 s	24.6-38.4 s	N
Fibrinogen	194 mg/dL	168-529 mg/dL	N
Coombs test	Negative		
ADAMTS13 activity	66%	40-130%	N
Serology			
Cytomegalovirus	IgG: negative; IgM: negative		No infection
EBV	IgG: positive; IgM: positive		Recent infection
Hepatitis B virus; hepatitis C virus	HBsAg: negative; anti-HBs: negative; anti-HBc: antibody to hepatitis B core antigen; anti-HCV: negative		No infection
Human immunodeficiency virus	Anti-HIV 1 and 2: negative		No infection

She was given 0.8 g/kg/day of intravenous immunoglobulin (IVIG) and 1 mg/kg of methylprednisolone for two days, as well as antibiotic therapy to prevent peritonitis, and hormonal therapy to prevent severe menorrhagia. As seen in Table 2, platelets rose to 43x10^9^/L 24 hours after the beginning of IVIG infusion, but hemoglobin lowered to 7.1 g/dL, requiring packed red blood cell transfusion. Control ultrasonography documented a significant decrease of the peritoneal effusion by D4, with complete resolution in subsequent evaluations. A gradual rise in platelet count and hemoglobin normalization was observed, achieving hemoglobin of 13 g/dL and 154x10^9^/L platelets with no further therapy by D12. The girl was discharged, maintaining regular clinical and laboratory surveillance. In the third week, she was given an oral prednisolone course (initial dose of 1 mg/kg/day) after her platelet count dropped to 27x10^9^/L, with a very good response (platelet count of 184x10^9^/L one week after), allowing its uneventful weaning off and discontinuation. After 12 months, she remains asymptomatic, with no medication, and platelet count stable above 200x10^9^/L. Further investigation, including antinuclear antibodies, anti-dsDNA antibodies, extractable nuclear antigen antibodies, immunoglobulins, direct Coombs test, Helicobacter pylori test, and screening of Von Willebrand disease, was negative.

**Table 2:** Evolution of hematological parameters in serial evaluations conducted after the start of therapy MCH: mean corpuscular hemoglobin; MCHC: mean corpuscular hemoglobin concentration; MCV: mean corpuscular volume; RDW: red blood cell distribution width; WBC: white blood cell

Laboratory test	Reference range	Days after start of therapy							
		1	2	3	12	19	26		12 months
WBC count	4.5-13.0x10^9^/L	7.3	7.4	6.3	5.8	6.5	12.7		5.9
Neutrophile	1.8-8.0x10^9^/L	4.8	5.3	2.6	2.3	3.8	8.8		3.6
Lymphocyte	1.5-5.2x10^9^/L	2.0	1.7	3.0	2.7	1.8	2.9		1.9
Monocyte	0.1-1.0x10^9^/L	0.5	0.3	0.5	0.5	0.5	1.0		0.3
Eosinophil	0.02-0.65x10^9^/L	0.01	0.06	0.04	0.11	0.26	0.02		0.12
Basophil	0.0-0.2x10^9^/L	0.05	0.09	0.14	0.14	0.13	0.05		0.06
Erythrocytes	4.1-5.1 x10^12^/L	2.27	2.78	3.39	4.43	4.11	4.44		4.14
Hemoglobin	12.0-16.0 g/dL	7.1	8.7	10.6	13.7	12.7	13.9		12.6
Hematocrit	36-46 %	20.4	25.2	31.4	39.8	36.6	38.5		37.4
MCV	78-102 fL	90	90.6	92.6	89.8	89.1	86.7		90.3
MCH	25-35 pg	31.3	31.3	31.3	30.9	30.9	31.3		30.4
MCHC	31-37 g/dL	34.8	34.5	33.8	34.4	34.7	36.1		33.7
RDW	11.5-15.0	13.3	13.5	14.6	12.4	12.1	12.1		12.1
Platelets	160-400 x10^9^/L	43	109	163	154	27	184		309
Observation		Red blood cell transfusion				Prednisolone course started			

## Discussion

ITP is characterized by the production of autoantibodies directed against platelet membrane antigens and their subsequent destruction [1,2,4,6]. It is more common in preschool age and in boys, but there is also a smaller peak in adolescence, characterized by a female predominance and higher prevalence of chronicity [1,3,6-8]. In approximately 60% of cases, there is a history of preceding viral illness, usually within the past month, EBV being one of the most commonly identified viruses, like in our case [1,6,7]. Vaccination against measles, mumps, and rubella is also associated with an increased risk, occurring in approximately 2.6 cases per 100,000 doses [1,6].

The symptoms are typically mild, and the child/adolescent is usually hemodynamically stable, with good appearance and no systemic signs and symptoms [1,6]. Other causes of thrombocytopenia should be considered if systemic symptoms are present, if there is a prior personal or family history of bleeding or if enlargement of lymph nodes, liver, or spleen is found [1]. Patients can also be asymptomatic, and the incidence of the disease is most likely underestimated [1].

The more common bleeding symptoms are cutaneous (“dry purpura”), like petechiae, purpura, or bruising, followed by mucosal (“wet purpura”) and, less frequently, menstrual, gastrointestinal, and urinary bleeding. Severe signs and symptoms are rare, developing in approximately 3% of children with ITP, and are related, among others, to the presence of severe thrombocytopenia (platelet count of <10x10^9^/L) [1,2,6,8].

To our knowledge, there are only a few reported cases of intra-abdominal bleeding in ITP, and these are usually related to ruptured cysts/follicles [2-4,8,9]. In this case, the source of bleeding was never identified, and she was on week four of her menstrual cycle, but the diagnosis of ruptured follicle/cyst cannot be excluded.

Thrombocytopenia (platelet count: <100x10^9^/L) is often the only abnormality detected, except in cases where significant bleeding leads to anemia. Any other laboratory findings should prompt the consideration of alternative causes [1,4,6,7,9]. In our case, the presence of anemia and abdominal pain prompted an imaging investigation, which led to the diagnosis of hematoperitoneum. In our case, anemia probably had an acute onset, in response to severe and recent peritoneal hemorrhage as the anemia was normocytic, and not microcytic with low iron stores as one would expect if the blood loss was chronic or longstanding. The normal findings obtained from additional investigations performed, coupled with the excellent response to therapy, strongly support this hypothesis over other potential diagnoses.

Supportive measures for ITP are aimed at avoiding bleeding events and rely on restriction from activities with a higher risk of bleeding/traumatic injury, avoidance of antiplatelet and anticoagulant medications (including ibuprofen, unless it is truly necessary), and control of bleeding manifestations, like menorrhagia and epistaxis [5,7]. ITP is generally a self-limiting benign disorder and patients with mild or no bleeding symptoms do not generally require admission to the hospital and can be managed in an ambulatory setting with only these measures and follow-up within 24 to 72 hours [2,5,6,10]. In cases of life-threatening bleeding, it is recommended to initiate immediate combination therapy consisting of platelet transfusion, intravenous corticosteroid, and IVIG. In severe but non-life-threatening hemorrhage, such as in our case, platelet transfusions are generally not necessary, and treatment with isolated corticosteroids or IVIG may be adequate [4,5]. Although IVIG is associated with more costs and side effects compared with corticosteroids alone, the need for a rapid increase in platelet count, given the intraabdominal bleeding, led to the use of IVIG in our case [3,5,6]. Because the platelet count dropped to less than 30x10^9^/L at two to three weeks (consistent with the known half-life of IVIG), we opted to start her on prednisolone as the initial presentation was severe bleeding and we were not able to identify the intraabdominal bleeding source. Due to the good and sustained response, prednisolone was successfully weaned off in eight weeks. She maintained a normal platelet count ever since. Surgical management of the bleeding is rarely needed, like in this case [3,9,11].

The prognosis of ITP is excellent, especially in children, with most patients recovering within three to six months from the time of presentation, regardless of instituted treatment [1,6,7,9]. In this case, the girl remained asymptomatic, with no medication after the short course of prednisolone.

## Conclusions

ITP is a relatively common childhood disease characterized by isolated thrombocytopenia. It typically presents with sudden mild to moderate mucocutaneous bleeding, and the prognosis is generally good with high rates of complete remission. However, severe complications, although rare, can occur and should be promptly diagnosed and treated to prevent mortality. In this case report, we present the case of a 14-year-old ITP patient with spontaneous severe peritoneal hemorrhage that required prompt diagnosis and treatment, highlighting the importance of recognizing and managing such a rare presentation in ITP.
